# The Involvement of *hybrid cluster protein 4*, *HCP4*, in Anaerobic Metabolism in *Chlamydomonas reinhardtii*

**DOI:** 10.1371/journal.pone.0149816

**Published:** 2016-03-01

**Authors:** Adam C. Olson, Clay J. Carter

**Affiliations:** 1 Integrated Biosciences Graduate Program, University of Minnesota, Duluth, MN, 55812, United States of America; 2 Department of Plant Biology, University of Minnesota, Saint Paul, MN, 55108, United States of America; Arizona State University, UNITED STATES

## Abstract

The unicellular green algae *Chlamydomonas reinhardtii* has long been studied for its unique fermentation pathways and has been evaluated as a candidate organism for biofuel production. Fermentation in *C*. *reinhardtii* is facilitated by a network of three predominant pathways producing four major byproducts: formate, ethanol, acetate and hydrogen. Previous microarray studies identified many genes as being highly up-regulated during anaerobiosis. For example, *hybrid cluster protein 4* (*HCP4*) was found to be one of the most highly up-regulated genes under anoxic conditions. Hybrid cluster proteins have long been studied for their unique spectroscopic properties, yet their biological functions remain largely unclear. To probe its role during anaerobiosis, *HCP4* was silenced using artificial microRNAs (*ami-hcp4*) followed by extensive phenotypic analyses of cells grown under anoxic conditions. Both the expression of key fermentative enzymes and their respective metabolites were significantly altered in *ami-hcp4*, with nitrogen uptake from the media also being significantly different than wild-type cells. The results strongly suggest a role for *HCP4* in regulating key fermentative and nitrogen utilization pathways.

## Introduction

*Chlamydomonas reinhardtii* is a predominantly soil dwelling microalgae found globally [1] that has long been used as a model system for studying photosynthesis, nutrient deprivation, flagellar function, and H_2_ production [2]. Interest in *C*. *reinhardtii* as model organism for biofuel production has been renewed in recent years due to: 1) the discovery of its ability to perform anaerobiosis in the light, 2) its rapid growth rates compared to terrestrial plants, and 3) development of ‘omics’ based approaches to elucidating metabolic pathways, including the development of genetic manipulation techniques, which can be used for the optimization of metabolic processes [3,4]. The generation of stable mutants in *C*. *reinhardtii* has traditionally been achieved by random genomic integration [4]. This approach is cumbersome and requires the screening of thousands of mutants using suitable phenotypic criteria or extensive DNA analysis. Recently, tools have been developed that enable targeted gene disruption through the use of artificial microRNAs (amiRNAs) [5,6] and CRISPR/Cas9 technologies [7].

### Fermentation pathways in *C*. *reinhardtii*

**Fig 1:**
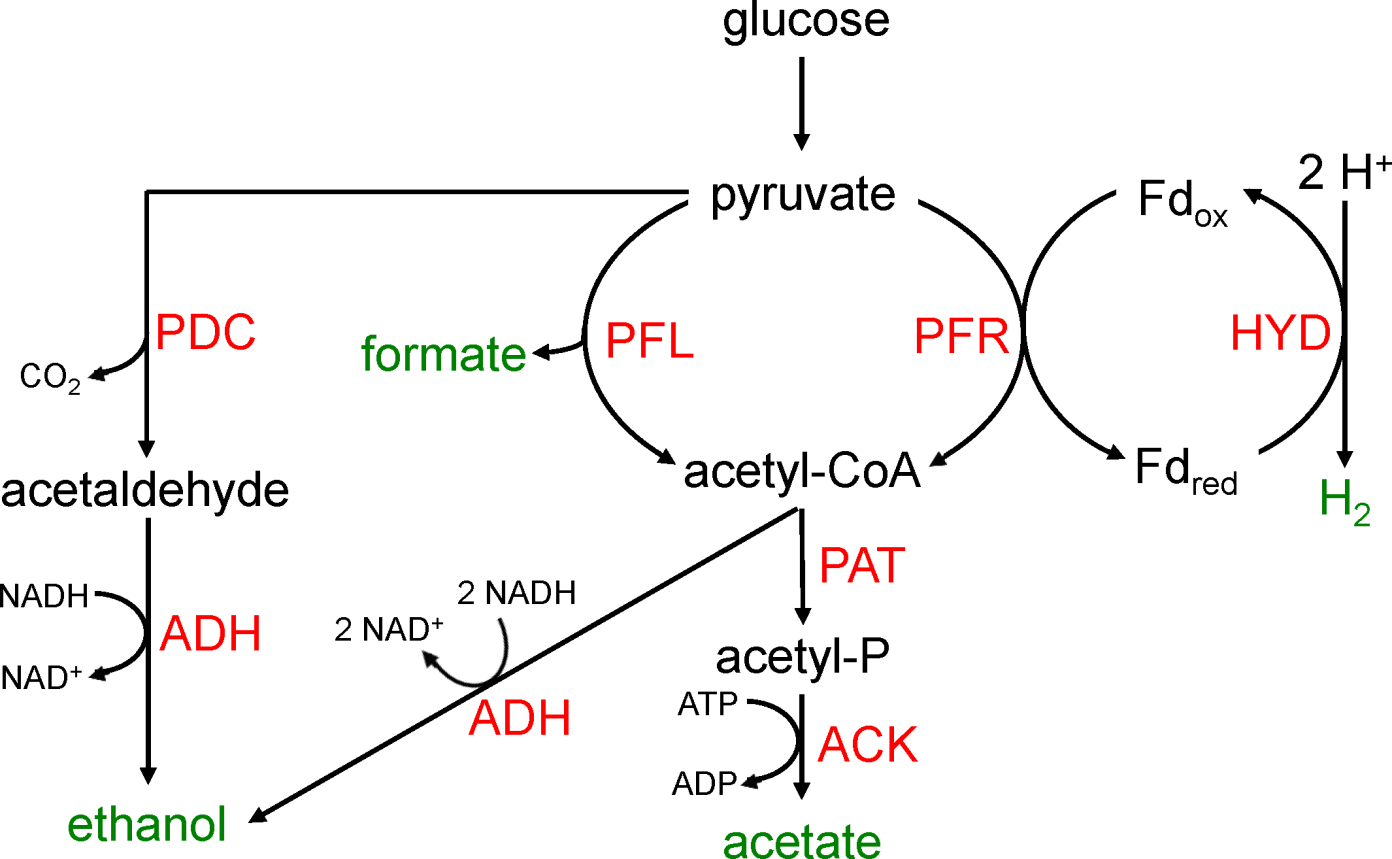
Major fermentation pathways of *Chlamydomonas reinhardtii*. Following glycolysis, pyruvate is further broken down to acetyl-CoA by pyruvate formate lyase (PFL) and pyruvate ferredoxin oxidoreductase (PFR). Acetaldehyde is formed by pyruvate decarboxylase (PDC) from pyruvate. Hydrogenase (HYD) oxidizes reduced ferredoxin to form H_2_ gas. Ethanol is formed from acetaldehyde and acetyl-CoA via Alcohol dehydrogenase (ADH). Acetate is formed from acetyl-CoA via phosphoacetyl transferase (PAT) and acetate kinase (ACK). Lactate is formed via aactate dehydrognenase (LDH). Modified from [2].

It is apparent that *C*. *reinhardtii* has evolved a diverse set of metabolic pathways to deal with the periods of anoxia it experiences in nature. Anoxia can be induced in the laboratory by placing sealed cultures in the dark, sparging oxygen from cultures (e.g. bubbling N_2_), or by placing cells in sulfur-free media and growing them in light (S is required for photosynthetic O_2_ evolution). Under the latter conditions, O_2_ uptake via respiration overcomes O_2_ production via photosynthesis leading to anoxia. As illustrated in Fig 1, fermentation in *C*. *reinhardtii* follows glycolysis by the breakdown of pyruvate. Six fermentation products are observed during darkness-induced fermentation: H_2_, CO_2_, acetate, ethanol, formate, and glycerol [8,9]. However, the main products of darkness-induced fermentation (anaerobiosis) are formate, acetate, and ethanol in a 2:1:1 ratio, with H_2_ and CO_2_ given off as minor byproducts [9,10]. A fermentation ratio of 2:2:1 of the respective metabolites has also been reported [2,8], though this discrepancy could be due to different culture conditions and strains of algae used in the studies [1].

*C*. *reinhardtii* is unique among eukaryotes in that it contains four enzymes used in pyruvate fermentation, including: pyruvate formate lyase (PFL), pyruvate ferredoxin oxidoreductase (PFR), lactate dehydrogenase (LDH), and pyruvate decarboxylase (PDC).

In addition to the four fermentative enzymes mentioned above, *C*. *reinhardtii* also expresses [FeFe] hydrogenases (HYD) and associated maturation proteins, which are rarely found in eukaryotes [1,2]. Hydrogen production in green algae was first demonstrated in 1942 by Hans Gaffron [11] and is facilitated in *C*. *reinhardtii* by reversible [FeFe] hydrogenases, of which there are two isoforms (HYDA1 and HYDA2) bound to the photosynthetic apparatus by ferredoxin [11]. H_2_ production takes place in strictly anaerobic environments, as HYD transcription and enzyme stability is severely compromised in the presence of oxygen (≈3% O_2_) [12]. Algal hydrogenases show high similarity to hydrogenases found in strict anaerobes, fungi and protists [13], but is directly reduced by ferredoxin, unlike other hydrogenases that rely on putative electron relays comprised of FeS clusters, either [2Fe2S] or [4Fe-4S] [13]. *HYD* expression is induced 100-fold upon darkness-induced anaerobiosis, though starchless mutants show attenuated hydrogenase expression suggesting other transcriptional regulators other than O_2_ [2,14,15]. This is likely due to the fact that dark fermentative H_2_ production pathway is directly coupled to starch catabolism [8,9]. In this pathway the oxidation of pyruvate is directly coupled to the reduction of ferredoxin by PFR while producing acetyl-CoA and CO_2_, with HYD then oxidizing the reduced ferredoxin forming H_2_. However, H_2_ is a very minor fermentation product during darkness-induced anaerobiosis [9,10].

### Hybrid cluster protein 4

The low H_2_ output observed during darkness induced anaerobiosis leads to questions regarding the presence of limiting steps or pathways competing with HYD for reduced ferredoxin. For example, *hybrid cluster protein 4* (*HCP4*, Cre09.g391650) displays a rapid and large increase in expression during darkness-induced anaerobiosis [2]. There are four HCP family members encoded by the *C*. *reinhardtii* genome, but only *HCP4* appears to be highly upregulated during anoxia [2]. Similar to HYD, HCP4 is an iron sulfur protein containing two subunits, a [4Fe-4S]2+/1+ or [2Fe-2S]2+/1+ and [4Fe-2S-2O], the so-called “hybrid cluster” [16]. The binding motif of [4Fe-4S] and [2Fe-2S] observed in HCP4 shows unique spacing of conserved cysteines making it similar to HCPs found in strict anaerobes [2]. Although this family of proteins has been studied extensively on a structural basis, its physiological role is not fully understood. *E*. *coli* HCP was found to be induced by hydrogen peroxide and is believed to play a role in oxidative stress defense [16]. *E*. *coli* HCP also shows up-regulation upon addition of nitrate or nitrite to the media, and purified HCP displays hydroxylamine reductase activity, reducing hydroxylamine to ammonia [17]. Further, hydroxylamine production was shown to require ferredoxin in *Clostridium pasteurianum* [18]. Due to these findings, it has been proposed that HCP4 in *C*. *reinhardtii* could oxidize reduced ferredoxin, thereby directly competing with HYD for electrons and thus lowering potential H_2_ yield [2].

### Nitrogen metabolism in *C*. *reinhardtii*

In *C*. *reinhardtii*, nitrate and ammonium are the predominant sources for nitrogen assimilation [19,20]. Nitrate is converted into nitrite in the cytoplasm by the enzyme nitrate reductase (NR) and is further transported into the chloroplast by NAR1 nitrite transporters [19,20]. In the chloroplast nitrite is converted to ammonium via the ferredoxin-dependent nitrite reductase (NiR) and is incorporated into L-glutamate via the glutamine synthetase/glutamate synthase cycle (GS/GOGAT) [19,20]. Light/dark cycles have been found to regulate NR activity with nitrate and nitrite uptake being highest in the light and lowest in the dark [21].

### Purpose of this study

Cumulatively, the extraordinary collection of fermentation pathways makes *C*. *reinhardtii* especially well adapted to anaerobiosis, and is able to produce multiple biofuels such as triacylglycerols, ethanol and H_2_ [22]. These fermentation products, along with the ability of *C*. *reinhardtii* to grow quickly to very high biomass densities in environments that will not compete with food stocks, makes it an excellent candidate for the development of biofuels. Indeed, *C*. *reinhardtii* and other microalgae have been the focus of global research into biofuel production for decades [23]. Despite this effort, the pathways involved in the diverse fermentation metabolism of *C*. *reinhardtii* are not fully understood. A further understanding of the interactions between the various fermentation pathways active in anaerobic *C*. *reinhardtii* will yield more precise targets in the effort to engineer better microalgae strains for biofuel production.

It is also clear that *C*. *reinhardtii* has as a diverse set of enzymes and pathways that are utilized in the nitrogen uptake and assimilation. Somewhat surprisingly the uptake of nitrogen has not been widely studied during anaerobic conditions. Ferredoxin plays a large role in nitrogen metabolism at the NiR, Fd-GOGAT, and possibly the NR stages, and it is known that darkness inhibited electron flow around PSI and PSII will slow nitrate, nitrite and ammonia uptake [24]. However the interplay between anaerobic energy production and anaerobic nitrogen metabolism has not been elucidated. HCP4 may be associated with anaerobic nitrogen metabolism based on the high upregulation of HCP4 during anaerobiosis and the hydroxylamine reductase activity observed in related proteins, as well as its hypothesized ability to oxidize reduced ferredoxin.

The purpose of this study was to use *C*. *reinhardtii* as a model system to investigate the role of *HCP4* on the interactions of various fermentation pathways in darkness-induced anaerobiosis. A further understanding of regulatory networks coordinating metabolic flux in *C*. *reinhardtii* is paramount in developing informed metabolic engineering strategies to boost biofuel production.

## Materials and Methods

### Algal strains and standard growth conditions

*Chlamydomonas reinhardtii* type cc425 arg2 cw15 sr-u-2-60 mt+ (referred to as cc425 or wild-type hereafter) was used as the wild-type background for all studies. All cultures were grown mixotrophically in Tris Acetate Phosphate (TAP) media [1] under 12 hr day / 12 hr night cycles with shaking at 120 rpm. During growth, wild-type strains were supplemented with 100 μg/ml arginine. Cultures were illuminated with a photosynthetic photon flux of 150 μmol m^-2^ s^-1^ and temperature of 23°C.

### Generation of amiRNA vectors

**Fig 2:**
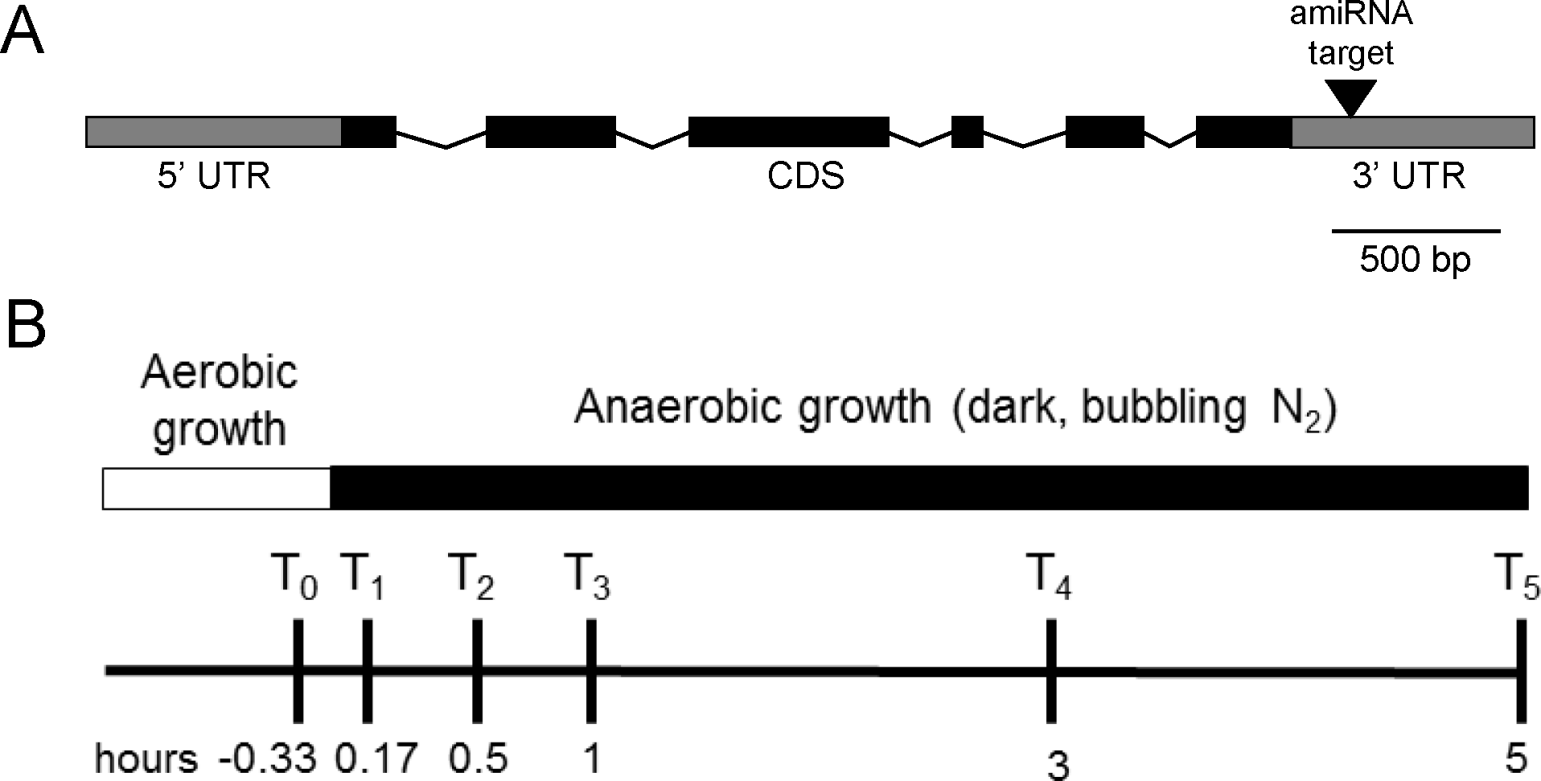
Experimental design for silencing HCP4 and induction of anaerobiosis. (A) Schematic of *HCP4* gene structure (Cre09.g391650), which was targeted in the 3’ UTR for degradation using a pChlamiRNA 2 vector (Molnar et al. 2009). (B) Schematic of time points of subsamples taken from anaerobic cultures. Cultures grown aerobically in TAP media were pelleted, resuspended in 28mM HEPES, pH 7.5 buffer (represented by far left of white box), and then allowed to acclimate for 50 minutes in the light with shaking prior to induction of anaerobiosis. Samples were taken from anaerobic cultures at 0.33 hours before anaerobic induction and then at 0.17, 0.5, 1, 3, and 5 hours post-anaerobiosis.

The vector pChlami2 was obtained from the *Chlamydomonas* resource center (University of Minnesota) and prepared according to Molnar et al. [5]. amiRNA inserts were generated for HCP4 using WMD (web based microRNA designer) version 3 (http://wmd3.weigelworld.org/cgi-bin/webapp.cgi?page=Home;project=stdwmd). A schematic of *HCP4* gene structure and the location of amiRNA targeting are indicated in Fig 2A. *HCP4* (XM_001694402) mRNA sequence was used to generate an appropriate amiRNA insert. Ninety nucleotide long oligonucleotides (S1 Table) were synthesized by Integrated DNA Technologies (IDT, Coralville, IA). Inserts were resuspended to a final concentration of 100 μM. To anneal the insert oligos, 10 μl of forward and reverse insert oligos were mixed with 20 μl 2X annealing buffer (20mM Tris pH 8.0, 2mM EDTA, 100mM NaCl). The mixture was boiled for five minutes and gradually cooled overnight. The double stranded insert was purified using Qiagen PCR clean up kit (Qiagen, Venlo, Netherlands). The insert was phosphorylated with using Promega T4 Polynucleotide Kinase (Promega Corp., Madison, WI, USA). The vector pChlami2 was digested with *Spe*I and dephosphorylated with calf intestinal alkaline phosphatase (CAIP). The dephosphorylated vectors were purified using Qiagen PCR clean up kit. The phosphorylated insert was then cloned into the vectors using Promega T4 DNA ligase (Promega Corp., Madison, WI, USA). Mach1 *E*. *coli* were transformed by electroporation with the vectors and plated on 150 μg/ml ampicillin LB agar plates.

Individual *E*. *coli* colonies were selected and grown in LB broth at 37°C and DNA was extracted via Qiagen miniprep kit (Qiagen, Venlo, Netherlands). PCR reactions were performed to locate transformed colonies containing the vector and insert in the correct orientation using primers AmiRNAprec_for_ (5’-GGTGTTGGGTCGGTGTTTTTG-3’) and Spacer_rev_ (5’-TAGCGCTGATCACCACCACCC-3’) were used with Promega GoTaq Green Master Mix according to the manufacturer’s instruction (Promega Corp., Madison, WI, USA). Candidate constructs containing the insert in the correct orientation were sequenced at the University of Michigan Sequencing Core (Ann Arbor, Michigan).

### *C*. *reinhardtii* transformation

*C*. *reinhardtii* strain cc425 was stably transformed with the vector pChlami2 containing the HCP4 amiRNA insert using a modified Kindle’s glass bead method [25]. Wild-type cc425 cells were re-suspended to a density of 1x10^6^ cells/ml. 300 μl of cells, 100 μl 20% polyethylene glycol (PEG), 2 μg HCP4 amiRNA vector, and 300 μg glass beads (0.5mm diameter) were added to a 1.5 ml microcentrifuge tube and vortexed on high for 30 seconds. 150 μl of cells were plated on a 1.5% TAP agar plates and incubated. Multiple independent transformants growing on TAP were selected and the sequence verified at the University of Michigan sequencing core (Ann Arbor, Michigan).

### Induction of anaerobic conditions

Liquid cultures were initiated by inoculating 500 ml of TAP with wild-type and *HCP4*-silenced cells (hereafter termed *ami-hcp4*). Cultures were grown for four days under 12 hour light-dark cycles, illuminated with a photosynthetic photon flux of 150 μmol m^-2^ s^-1^ and temperature of 23°C. Following incubation cells were counted via hemocytometer and 160x10^6^ cells (both wild-type and *ami-hcp4*) were pelleted and washed with 25 ml HEPES buffer (28mM, pH 7.5). Pellets were then resuspended in 40ml HEPES buffer (28mM, pH 7.5) to a final concentration of 4x10^6^ cells/ml in a 50ml conical tube and incubated in the light for 1 hour. Following incubation, cell vitality was assayed by noting swimming cells in each culture. The tubes were wrapped in foil, with Parafilm loosely applied to the tops. N_2_ gas was bubbled through the cultures and light excluded by placing a foil covered box over the cultures. Dissolved oxygen was assayed using a Clark-type electrode following 10 minutes of N_2_ bubbling to ensure anoxic conditions were achieved. As illustrated in Fig 2B, subsamples were collected from cultures that were resuspended in 28 mM HEPES, pH 7.5 buffer at T_0_: following 40 minutes incubation in light under aerobic conditions, which was equivalent to 20 minutes before (-0.33 hours) induction of anaerobiosis, T_1_: 10 minutes after the initiation of anaerobiosis (following 10 minutes N_2_ bubbling in dark, 0.17 hours), T_2_: 0.5 hours post-anaerobiosis, T_3_: 1 hour post-anaerobiosis, T_4_: 3 hours post-anaerobiosis, T_5_: 5 hours post-anaerobiosis. Cell viability and motility were verified at each experimental time point.

### Gene expression analyses

Trizol (Invitrogen, San Diego, California) was used to extract RNA from the frozen pellets. Pellets were resuspended in 1ml Trizol by pipetting and samples were then incubated at room temp for 5 minutes; 200 μl of chloroform was then added and tubes shaken by hand for 15 seconds then incubated at room temp for 2 minutes. Samples were spun at 11,000 g for two minutes at 4°C. The upper aqueous phase was placed in a new tube, 0.5 ml of isopropyl alcohol was added and the tube was incubated at room temperature for 10 minutes. Samples were then centrifuged at 11,000 g for 10 minutes at 4°C. The supernatant was removed and replaced with 1ml of 75% ethanol and mixed gently by hand. The mixture was centrifuged at 7,000 g for five minutes at 4°C. The supernatant was removed and let air dry for five minutes. The RNA pellet was resuspended in 40 μl RNase-free H_2_O and incubated at 55°C for 10 minutes. RNA was quantified and integrity was verified by running 500 μg of RNA in a 2% agarose gel.

Reverse transcription was carried out using a Qiagen Quantitect Reverse Transcription kit and 500 μg RNA according to manufacturer’s instructions (Qiagen, Venlo, Netherlands). Real time PCR was performed using Rotor-gene SYBR Green PCR Kit (Qiagen, Venlo, Netherlands) and Corbett Research RG-3000 thermocycler (Qiagen, Venlo, Netherlands). One μl of cDNA was used for each reaction. Previously published primers were used to amplify 100–200 nucleotide regions of the following genes; Rack1, beta-tubulin, PDC, HYD, PFL, PFR, and HCP4 (S2 Table) [2]. Cycling parameters contained a melting step at 95°C for 10 minutes followed by 65 cycles of a 95°C (10 sec) melting step followed by a 60°C (15 sec) annealing/elongation step. Data were acquired on the FAM/Sybrgreen channel during the annealing/elongation step. A 10 minute step at 72°C ended the cycle. Relative expression was calculated using the comparative Ct Method (Applied Biosystems). Rack1 and beta-tubulin were used as constitutively expressed controls.

### Organic metabolite assays

The metabolites formate, ethanol and acetate were measured in the media using kits (formate; 10979732035, acetate; 10148261035, ethanol; 10176290035) from Boehringer Mannheim / r-biopharm, Darmstadt, Germany. These enzymatic assays measure sample dependent production of NADH. NADH was measured at 340nm for the ethanol and acetate assays using a Beckman DU 650 spectrophotometer (Beckman Coulter, Brea, CA). NADH was measured in the formate assay using a Nanodrop ND-1000 spectrophotometer (ThermoScientific, Waltham, MA). Supernatant fractions from each time point were used as samples and HEPES buffer used as the blank. Manufacturer instructions were followed with slight modifications. The ethanol reaction volume was reduced to 525 μl and 100 μl sample volume was used in each reaction. The formate reaction volume was reduced to 61 μl and 40 μl sample volume was used. The Acetate reaction volume was reduced to 537 μl using a sample volume of 100 μl. Results are reported as the average of quadruplicate samples for formate and ethanol and triplicate samples of acetate at each time point.

### Nitrogen uptake assays

Uptake of nitrate or ammonium was measured over a 24 hour period. Liquid cultures were initiated by spiking 500ml of TAP liquid with equal amounts of wild-type and *ami-hcp4* cells. Cultures were grown for four days under 12 hour light dark cycles, illuminated with a photosynthetic photon flux of 150 μmol m^-2^ s^-1^ and temperature of 23°C. Following incubation the cells density was measured using a hemocytometer and 160x10^6^ cells were pelleted and washed with ammonium solution (12mM Na-acetate, 28mM HEPES, 10mM NH_4_Cl) or nitrate solution (12mM Na-acetate, 28mM HEPES, 10mM KNO_3_). Cells were then resuspended in 40 ml of their respective ammonia or nitrate solutions at 4x10^6^ cells/ml. Cells were bubbled with N_2_ in the dark for ten minutes then capped and placed in the dark. Two ml subsamples were taken from the ammonium uptake experiment every two hours for 12 hours and then every 4 hours until 24 hours had elapsed. After being taken, samples were immediately centrifuged and separated into pellet and supernatant fractions. RNA was extracted from the four hour time point of the ammonia and nitrate uptake samples and silencing of *HCP4* was confirmed as described earlier.

Ammonium and nitrate remaining in solution were assayed using kits (ammonium; 11112732035, nitrate; 10905658035) from Boehringer Mannheim / r-biopharm, Darmstadt, Germany. Manufacturer’s directions were followed with slight modifications. Reaction volume for the ammonia and nitrate assays were reduced to 503.3 μl, and 508.33μl respectively. NADH was measured spectroscopically using a Nanodrop 2000c spectrophotometer (Thermo scientific, Waltham, MA).

## Results

Previous studied identified *HCP4* as one of the most highly upregulated genes during anaerobiosis [2]. To examine the role of *HCP4* in *C*. *reinhardtii* fermentation and nitrogen utilization, a construct encoding an amiRNA targeting the 3’ UTR of *HCP4* (Fig 2A) was generated and used to stably transform cc425 (wild-type) *C*. *reinhardtii*.

### Gene expression profiling

**Fig 3:**
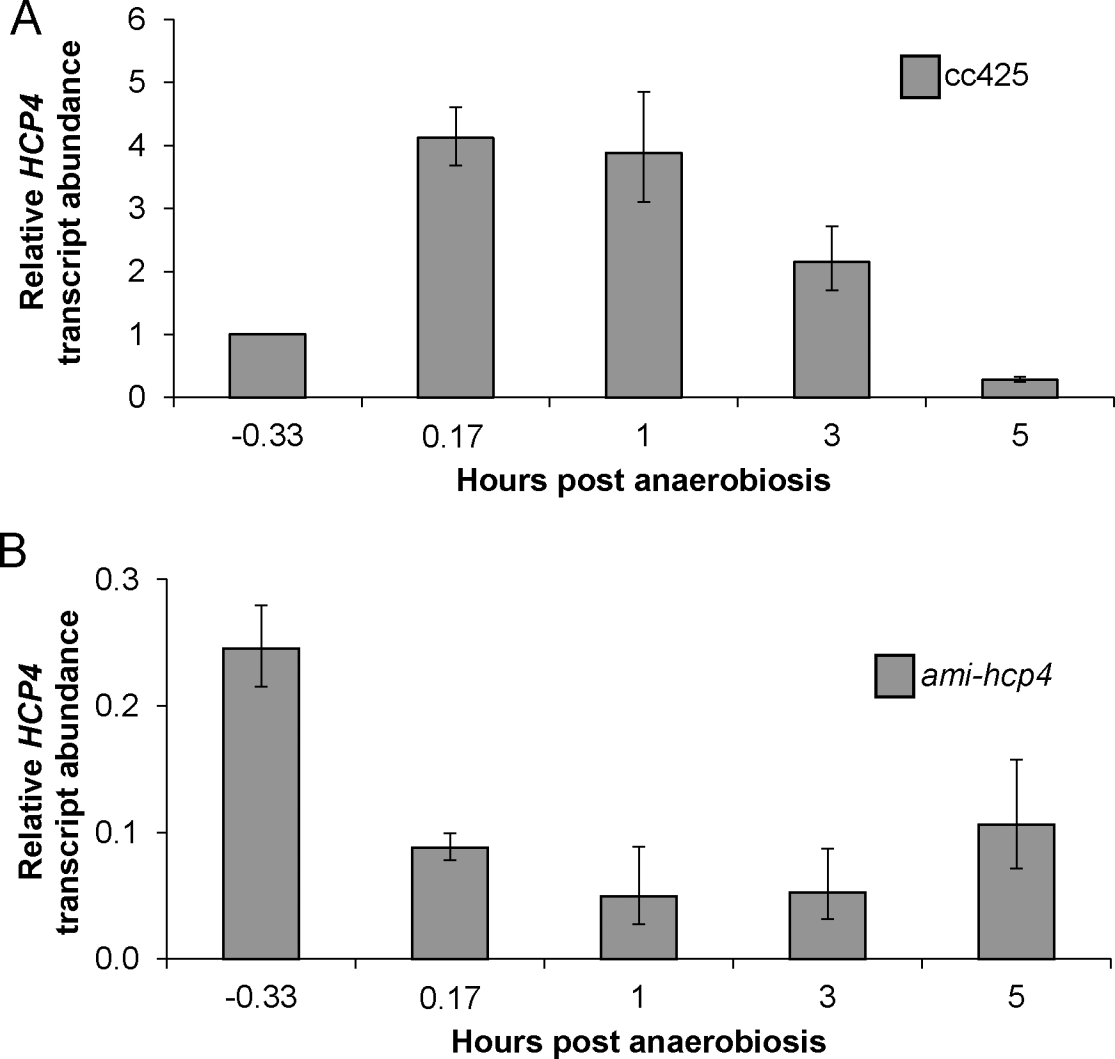
Analysis of *HCP4* transcripts under experimental conditions. (A) Induction of *HCP4* transcription in wild-type cells under darkness-induced anaerobic conditions. All post-anaerobiosis time points were significantly different than pre-anaerobiosis (data presented relative -0.33 hours in wild-type CC425, p<0.05). (B) The effect of amiRNA vector targeting *HCP4* on transcript levels under the same anaerobic conditions as wild-type cells. *HCP4* mRNA levels were strongly reduced in *ami-hcp4* relative to wild-type throughout the course of anaerobiosis. mRNA was extracted at the indicated time points and cDNA levels measured by qPCR in wild-type and *ami-hcp4* (data is presented as expression of *HCP4* in *ami-hcp4* relative to wild-type at each time point). *n* = 2 biological replicates with 3 technical replicates for both A and B.

Wild-type cc425 and *ami-hcp4* cells were grown in TAP media, pelleted, washed, and resuspended in 28mM HEPES buffer and subjected to five hours of anoxic conditions through growth in the dark while being bubbled with a constant stream of N_2_ gas. No gross phenotypic differences relative to wild-type were noted in *ami-hcp4* cell number, viability, or motility during either aerobic or anaerobic growth (in either TAP media or HEPES buffer). Subsamples were taken at -0.33, 0.17, 0.5, 1, 3, and 5 hours after induction of anaerobic conditions as outlined in Fig 2B. To confirm that *HCP4* expression was upregulated in wild-type cells grown under the selected anaerobic conditions, and that *HCP4* was indeed silenced in transgenic mutants (*ami-hcp4*), mRNA was extracted and evaluated by quantitative RT-PCR. Indeed, under our experimental conditions, *HCP4* transcription was significantly elevated within 10 minutes of induction of anaerobic conditions (Fig 3A), and remained elevated for at least 3 hours. However, *HCP4* expression was ~3-fold less than wild-type cells after 5 hours of growth under anaerobic conditions.

**Fig 4:**
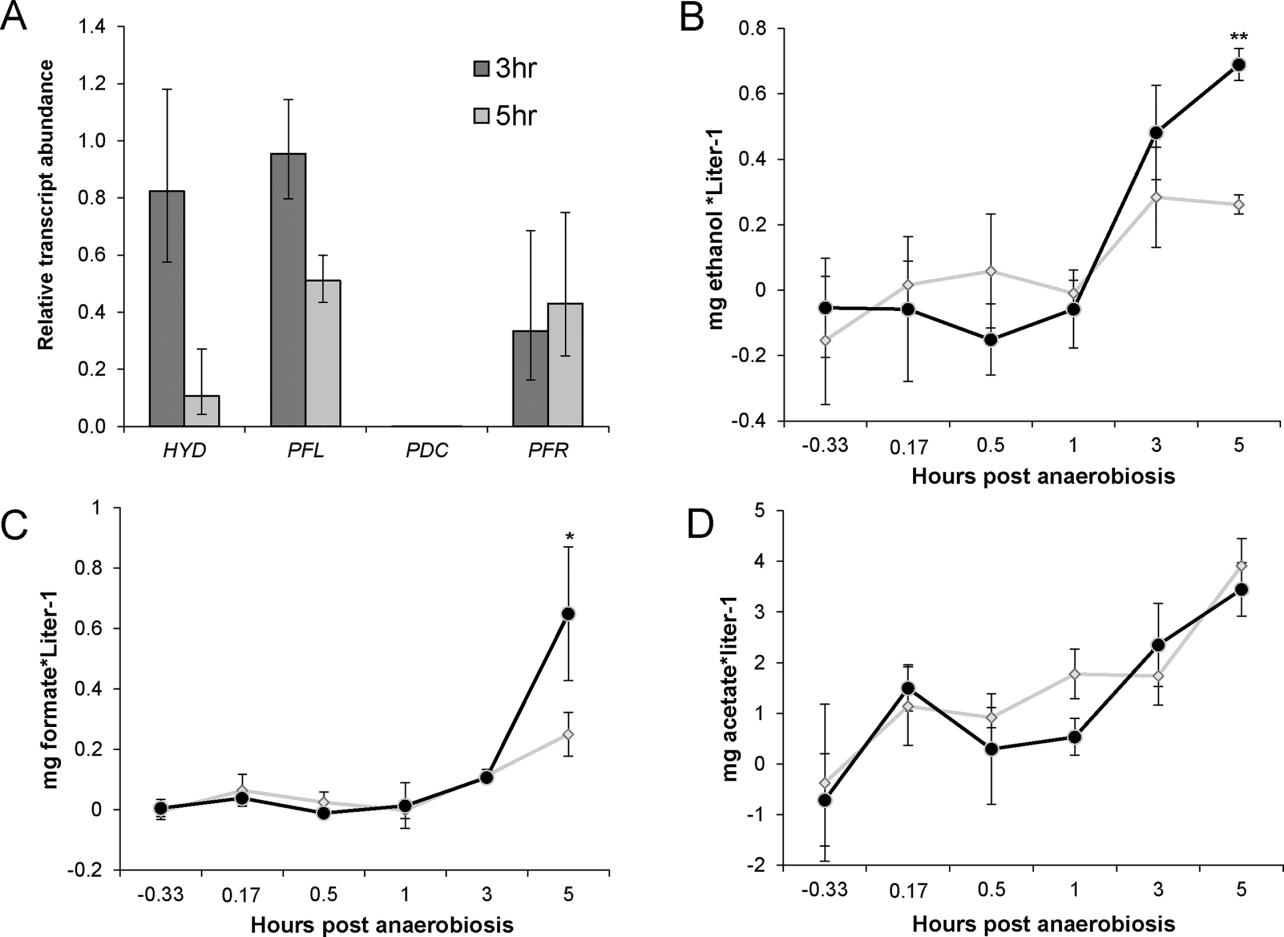
The effect of silencing *HCP4* on fermentation pathways. (A) Expression of key fermentation transcripts in ami-*hcp4* relative to wild-type CC425 at both 3 and 5 hours post-anaerobiosis; n = 3 biological replicates with triplicate technical replicates. (B) Ethanol, (C) formate, and (D) acetate accumulation during darkness-induced anaerobiosis in wild-type cc425 (gray diamonds) and *hcp4* (black circles). Cultures were resuspended in 28 mM HEPES, pH 7.5 at -1 hour post anaerobiosis and constantly bubbled with N_2_ in the dark starting at 0 hours post anaerobiosis. **p <0.01; * p<0.05; n = 4 biological replicates for ethanol and formate, n = 3 biological replicates for acetate.

Quantitative RT-PCR analyses also demonstrated *HCP4* transcript levels were significantly reduced in *ami-hcp4* at all time points (Fig 3B). Silencing of *HCP4* in *ami-hcp4* ranged from 0.24 relative transcript abundance (4-fold knockdown) at 0.33 hours pre-anaerobiosis to 0.05 relative transcript abundance (20-fold knockdown) at 1 hour post-anaerobiosis. Once silencing of *HCP4* was confirmed, the expression of other genes central to fermentation pathways, including *HYD*, *PFL*, *PDC*, and *PFR*, were investigated to elucidate the effects of silencing *HCP4* (Fig 4A). Relative transcript abundance was measured using the same cDNA synthesized from 3 and 5 hours post-anaerobiosis described above. At three hours post-anaerobiosis HYD displayed a slight decrease in expression, but this change was not statistically significant. At five hours, however, HYD showed a 10-fold decrease in expression relative to wild-type. Similarly, PFL was not significantly downregulated at 3 hours post-anaerobiosis, but at 5 hours showed a significant 2-fold decrease in expression. PFR expression was decreased at both the 3 and 5 hour time points, displaying 0.33 and 0.44 relative transcript abundance, or a 3-fold and 2.3-fold decrease in expression, respectively. Surprisingly, PDC levels showed negligible expression in *ami-hcp4* cells at both the three and five hour time points, which was confirmed in multiple independent transformants.

### Metabolite production during anaerobiosis

The downregulation of keys genes for several major fermentation pathways in *ami-hcp4* suggested that the metabolites from these pathways may also be reduced. The metabolites ethanol, formate and acetate were measured using enzymatic assays at all time points taken during the course of anaerobiosis. Production of ethanol was not significantly different between wild-type and *ami-hcp4* lines until 5 hours post-anaerobiosis (Fig 4B). At 3 hours post-anaerobiosis wild-type and *ami-hcp4* showed measureable excreted ethanol at mean concentrations of 0.28 and 0.48 mg*ml^-1^ respectively, yet these values were not statistically significant from each other. At 5 hours, wild-type and *ami-hcp4* production significantly diverge as *ami-hcp4* displayed an average 2.6-fold increase in excreted ethanol. Significant difference was calculated at five hours by Student’s unpaired t-test with equal variance (p = 0.0002).

Production of formate was not detectable until 3 hours post-anaerobiosis (Fig 4C). At 3 hours levels of formate excreted were not significantly different between wild-type and *ami-hcp4* which were 0.11 and 0.10 mg formate*liter^-1^ respectively. However, at 5 hours post-anaerobiosis production of formate significantly diverged between wild-type and *ami-hcp4*, with wild-type accumulating 0.25 mg formate*liter^-1^ and *ami-hcp4* accumulating 0.65 mg formate*liter^-1^. Thus *ami-hcp4* had a ~2.6-fold increase in formate accumulation at 5 hours post-anaerobiosis relative to wild-type. Significant difference was calculated at 5 hours by Student’s unpaired t-test with equal variance (p = 0.045).

Acetate accumulation during anaerobiosis was not significantly different at any time point between wild-type and *ami-hcp4* (Fig 4D). Both cultures showed acetate accumulation beginning at 0.17 hours post-anaerobiosis. Acetate accumulation progressively increased in both cultures until 5 hours post-anaerobiosis when wild-type and *ami-hcp4* accumulated an average of 3.9 and 3.4 mg acetate*liter^-1^ respectively.

As reported in Table 1, at 3 hours post-anaerobiosis wild-type accumulated the metabolites formate, acetate and ethanol at a 1:12:2.5 ratio. At the same time point *ami-hcp4* accumulated these metabolites at a 1:16:4.2 ratio. At 5 hours post-anaerobiosis wild-type accumulated the metabolites formate, acetate and ethanol at a ratio of 1:12:1, whereas *ami-hcp4* accumulated these metabolites at a ratio of 1:4:1.

**Table 1:** Mean metabolite totals from 3 and 5 hours after induction of anaerobiosis.

	Strain	Formate (mg)	Acetate (mg)	Ethanol (mg)	Molar ratio (formate:acetate:ethanol)
3 hour	cc425	0.11 ± 0.02	1.74 ± 0.56	0.28 ± 0.15	1:12:2.5
	*ami-hcp4*	0.11 ± 0.01	2.35 ± 0.82	0.48 ± 0.14	1:16:4.2
5 hour	cc425	0.25 ± 0.07	3.91 ± 0.53	0.26 ± 0.03	1:12:1
	*ami-hcp4*	0.65 ± 0.22*	3.45 ± 0.53	0.69 ± 0.05**	1:4:1

*p<0.05

**p<0.01 for *ami-hcp4* relative to cc425 (wild-type) at the respective time points.

### Nitrogen uptake assays

The expression of hybrid cluster proteins is up-regulated in *E*. *coli* cultures upon the addition of nitrate or nitrite [26]. Similarly, the only known biochemical function of any HCP from any species is that of hydroxylamine reductase in *E*.*coli* [17]. These findings suggest HCP4 may be involved in nitrogen metabolism in C. *reinhardtii*. Thus, nitrate and ammonium uptake were assayed in wild-type and *ami-hcp4* strains under anaerobic conditions.

**Fig 5:**
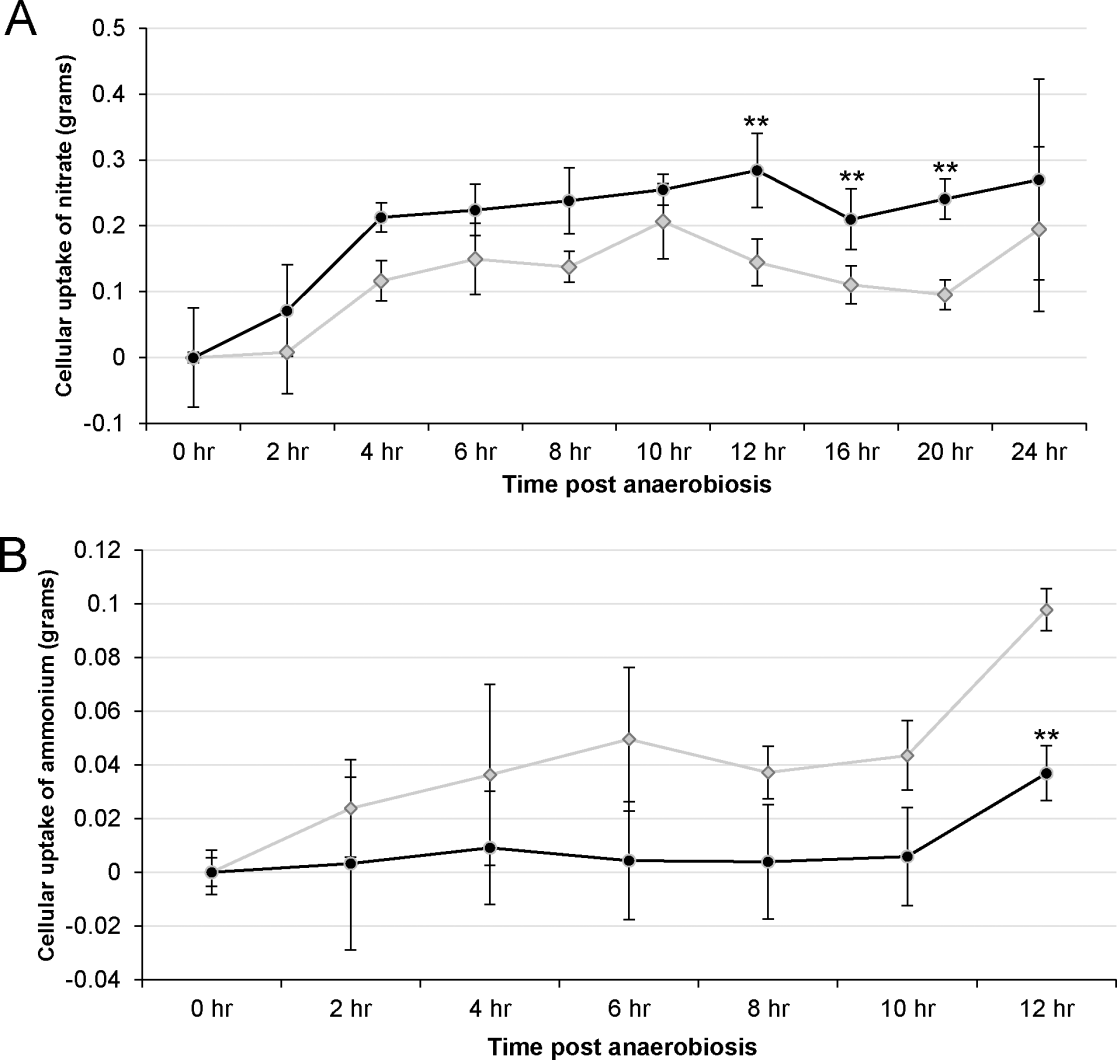
Nitrate and ammonium uptake during anaerobiosis. Nitrate and ammonium uptake were measured for 24 and 12 hours during anaerobiosis, respectively. Wild-type cc425 (gray diamonds) and *hcp4* (black circles). Cultures were grown in 10 mM HEPES, 10 mM ammonium, and 12 mM acetate solution. Cells were bubbled with N_2_ for 10 minutes and kept sealed in the dark. Subsamples were taken every two hours and nitrate and ammonium remaining in solution measured. ** t-test, p <0.01, n = 3 biological replicates for A and B.

Nitrate uptake in wild-type and *ami-hcp4* were similar to one another, but *ami-hcp4* had a consistently higher uptake of nitrate, with three out of ten time points (12, 16 and 20 hours post-anaerobiosis) being statistically different than wild-type (Fig 5A). Four hours post-anaerobiosis marked the first notable divergence in nitrate uptake with wild-type and *ami-hcp4* cells having uptaken an average of 0.12 and 0.22 grams of nitrate respectively. Twelve hours post-anaerobiosis marked the greatest divergence in nitrate uptake with wild-type and *ami-hcp4* having uptaken a mean of 0.14 and 0.28 grams of nitrate respectively. The trend in this data shows that cc425 nitrate uptake peaked at 10 hours post-anaerobiosis while *ami-hcp4* peaked at 12 hours.

Ammonium uptake was measured for only 12 hours under anaerobic conditions (Fig 5B) due to low viability of the cells in the later stages of the experimental conditions used. Wild-type and *ami-hcp4* ammonium uptake diverged at 8, 10, and 12 hour time points. At 8 hours post-anaerobiosis mean ammonium uptake in wild-type and *ami-hcp4* were 0.03 and 0.003 grams respectively. At 12 hours post-anaerobiosis ammonium uptake in wild-type and *ami-hcp4* were 0.1 and 0.06 grams of ammonium respectively. The general trend in Fig 5B shows relatively constant ammonium uptake in wild-type cells, but ammonium uptake in *ami-hcp4* did not take place until 12 hours post-anaerobiosis. A significant difference in ammonium uptake was calculated at 12 hours post-anaerobiosis by Student’s unpaired t-test with equal variance (p = 0.012).

## Discussion

*C*. *reinhardtii* has a uniquely diverse set of metabolic pathways that enable it to cope with a multitude of environmental situations including extended periods of anoxia. Recent metabolic and genetic studies have uncovered genes, pathways, and proteins that are up-regulated or activated during anaerobiosis [2,27,28]. The multiple pathways facilitating the continuation of glycolysis during anaerobiosis make *C*. *reinhardtii* fermentation one of the most diverse in the plant world and have been shown to be plastic in nature [29]. Being one of the most highly up-regulated genes during anaerobiosis in *C*. *reinhardtii*, HCP4 proves to be an interesting candidate for study as its physiological role in organisms has not been precisely determined, yet is activated during O_2_ deprivation in many prokaryotes. This study demonstrates that silencing of *HCP4* impacts multiple fermentation pathways, including changes in gene transcription, as well as metabolite flux and nitrogen uptake.

*HCP4* transcription in *ami-hcp4* was decreased consistently during anaerobiosis. The -0.33 hour time point showed a 4-fold knockdown, while one hour post-anaerobiosis a 20-fold knockdown was achieved. This increase in silencing level across time points in *ami-hcp4* compared to wild-type was likely due to the rapid induction of *HCP4* upon anaerobiosis as shown in Fig 3A. Knockdown of *HCP4* also led to dramatic differences in the expression of key genes for different fermentation pathways (Fig 4A). *HYD* and *PFL* transcripts showed no significant change in expression after 3 hours of anaerobiosis, but were dramatically reduced after 5 hours of anaerobiosis. *PFR*, which links pyruvate metabolism to ferredoxin, was shown to be consistently lower at 3 and 5 hours post-anaerobiosis. *PDC* expression, however, was also dramatically reduced at all time points. PDC transcript levels were also measured at earlier time points and in multiple independent transformants and found to be similarly silenced. Taken together, a general repression in gene expression for fermentation pathways was noted. These findings strongly suggest HCP4 is either directly or indirectly involved in regulating cellular responses to anonxia.

Metabolite assays also showed a significant difference between wild-type and *ami-hcp4*. As shown in Fig 4, the accumulation of ethanol and formate in the media began at 3 hours post-anaerobiosis. Significant differences in ethanol and formate accumulation between wild-type and *ami-hcp4* appeared at 5 hours post-anaerobiosis (Fig 4B and 4C). At 3 hours post-anaerobiosis *ami-hcp4* displayed a ratio of ethanol-to-formate production of ~4:1, although neither metabolite displayed significantly different accumulation between *ami-hcp4* and wild-type at this time point. This ~4:1 ratio of ethanol to formate production perhaps indicates that acetyl-CoA is being produced by both PFL and PFR and is being converted to acetate as well as ethanol. It is possible that enough PFR activity is still present even with the reduced transcript levels witnessed at this time point which contributes acetyl-CoA that is favorably converted to acetate.

**Fig 6:**
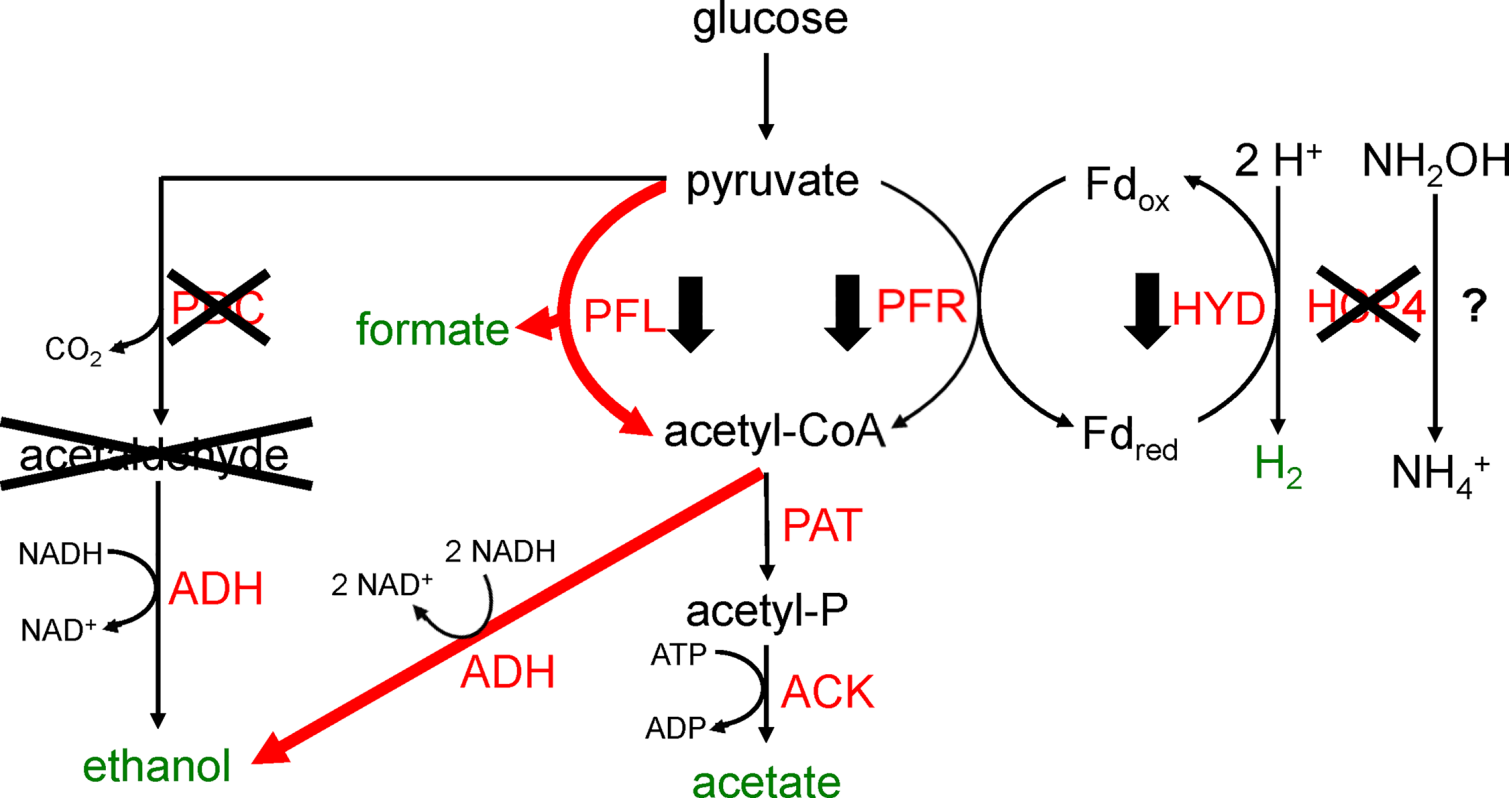
Summary of altered fermentation pathways upon HCP4 silencing. *PDC*, *PFL*, *PFR*, and *HYD* gene transcripts were significantly reduced in *ami-hcp4* relative to wild-type at five hours post-anaerobiosis. It is proposed that at five hours post anaerobiosis greater electron flux through PFL and ADH (red lines) leads to increased formate and ethanol production in *hcp4*. It is also possible that HCP4 may be competing with HYD for electrons from reduced ferredoxin in wild-type cells. A putative function for HCP4 as a hydroxylamine reductase is indicated. The effect of silencing *HCP4* on H_2_ production is unknown.

Interestingly, both formate and ethanol accumulation are roughly 2.6-fold higher in *ami-hcp4* than wild-type at five hours post-anaerobiosis, displaying a 1:1 ratio of production. Similarly, ethanol and formate production in wild-type occur at a roughly 2:1 ratio at three hours post-anaerobiosis and a ratio of 1:1 at five hours post-anaerobiosis. PFL facilitates the breakdown of pyruvate into formate and acetyl-CoA in a 1:1 ratio. If we assume that the down-regulation of *PFR* shown in *ami-hcp4* effectively blocks the breakdown of pyruvate by this pathway, and the more than anticipated acetate accumulation during fermentation in the media is due to excretion of acetate accumulated during mixotrophic growth, then these data suggest that the ethanol produced in *C*. *reinhardtii* at 5 hours post-anaerobiosis is facilitated exclusively by PFL in both wild-type and *ami-hcp4*. Conversely, at 3 hours post-anaerobiosis the ratio of ethanol-to-formate suggest that PFR is actively converting pyruvate into acetyl-CoA while reducing ferredoxin or acetyl-CoA produced by PFL is being converted to acetate. This shift in metabolic flux happens precisely when ethanol and formate start to accumulate in the media. It seems possible that the accumulation of metabolites may act as a regulator during anaerobiosis, possibly being detected directly, or by secondary signaling molecules such as pH or oxidative stress. Taken together, a general model of the response of fermentation pathway gene expression and metabolic flux is presented in Fig 6. At 5 hours post-anaerobiosis there is a general decrease in fermentation gene transcription, albeit with a hypothesized increase in electron flux through the PFL and ADH pathways.

One of the most startling findings of this study was the drastic down-regulation of *PDC* transcription. PDC is a cytoplasmic protein that is an integral component in yeast alcoholic fermentation. It has been shown that PDC does not participate highly in *C*. *reinhardtii* fermentation, but its activity and gene expression are both increased during anaerobiosis [2,29]. In yeast, PDC shows optimal activity in cells grown at a pH 6.0, but a decrease of only 7% in activity was shown when resuspended at a pH of 7.4, which is close to the pH of this experiment (7.5) [30]. PDC in maize is induced upon anaerobiosis only when cellular pH dropped from 7.4 to 6.8 [31]. Expression data in *C*. *reinhardtii* shows PDC up-regulation takes place in media at pH 7.0 and pH 7.3 [2,32]. Thus extracellular pH does not appear to similarly regulate PDC expression in *C*. *reinhardtii*. It is possible however that intracellular pH levels drop during anaerobiosis causing PDC to be activated. It is possible that an intracellular drop in pH is inhibited in *ami-hcp4*, thereby inhibiting PDC transcription. A potential mechanism of *HCP4* in controlling intracellular pH is currently unknown.

Acetate accumulation in wild-type and *ami-hcp4* cultures were not significantly different at any time point, and levels in both cell types increased steadily throughout the course of the experiment (Fig 4D). Acetate levels were significantly higher than ethanol and formate, which conflicts with previously published data stating the ratio of formate:acetate:ethanol should be roughly 2:1:1 or 2:2:1 [2,8,9]. While the data presented here offer a much different ratio of metabolic byproducts, differences in experimental procedures may account for this. In the Gfeller et al. [9] and Ohta et al. [8] studies, cells were grown autotrophically using CO_2_ as a carbon source, while also using different strains (F-60 and MGA 161, respectively). In this study however, acetate was used as a carbon source during mixotrophic growth. Thus when washed and resuspended in HEPES buffer before anaerobiosis, the cells possibly contained high internal stores of acetate (or starch leading to acetate production) which may have been excreted during anaerobiosis. Less clear are the differences in metabolite accumulation observed in this study and that from Mus *et al*. [2], which used the same strain and similar growth conditions. The only notable difference in growth conditions was that the cells in the current study were resuspended in 28 mM HEPES, pH 7.5 prior to induction of anoxia, whereas Mus *et al*. used 50 mM potassium phosphate (pH 7.0) supplemented with 3 mM MgCl_2_. It would be interesting to repeat the current study using alternative buffers during anoxic growth to determine how the culture conditions impact metabolite evolution.

HCP-family proteins have been implicated in nitrogen metabolism by studies in *E*.*coli*. *HCP* expression is up-regulated in *E*. *coli* upon addition of nitrate or nitrite [26], and purified HCP also has hydroxylamine reductase activity [17]. Hydroxylamine reductase catalyzes the reversible conversion of hydroxylamine to ammonia using ferredoxin as an electron donor [18]. HCP4 in *C*. *reinhardtii* shows moderate amino acid sequence similarity with the *E*.*coli* HCP (which has demonstrated hydroxylamine reductase activity), with 41.7% identity and 59.1% similarity (S1 Fig). Our study also shows a potential effect of HCP4 on nitrogen utilization during anaerobiosis. *ami-hcp4* showed increased nitrate uptake, but decreased ammonia uptake compared to wild-type (Fig 5). It has been hypothesized that nitrite reductase requires ferredoxin to convert nitrite to ammonium [33]. The data presented here shows nitrate uptake being enhanced in *ami-hcp4*, which may indicate HCP4 is also competing with nitrite reductase for electrons from ferredoxin. It is interesting that significant changes in ammonium uptake were not present in *ami-hcp4* until 12 hours post-anaerobiosis. This lag in ammonium uptake in *ami-hcp4* may indicate that HCP4 has a primary role responsible for ammonium uptake in *C*. *reinhardtii*. Alternatively, it is certainly possible that the observed changes in N uptake in *ami-hcp4* may be due to secondary effects of silencing *HCP4*. Clearly, future experiments examining hydroxylamine and nitrite reductase activities (as well as N utilization with more temporal resolution) in both mutant and wild-type cultures are warranted.

Taken together these data suggest a preliminary model of HCP4’s role in the cell during darkness-induced anaerobiosis. The sequence similarity of *HCP4* in *C*. *reinhardtii* to HCP in *E*. *coli*, and the increase in nitrate uptake when HCP4 is silenced, suggest that HCP4 is oxidizing reduced ferredoxin. With this working hypothesis, the other data collected in this experiment can be analyzed and a preliminary model produced. The silencing of *HCP4* may cause PFR to be downregulated due to the reduced electron flow out of ferredoxin. Whether or not HCP4 competition for reduced ferredoxin has an impact on H_2_ output is unknown. In this model HCP4 acts as a “release valve” for electrons from ferredoxin. A decreased reduction of pyruvate due to the depressed transcription of PFR may lead to a decrease of acetyl-CoA, which in turn would cause a decrease in NAD^+^ and ATP production through the PAT/ACK or ADH pathways (Fig 1 and Fig 6). This loss in NAD^+^ and/or ATP production would be compensated for by the accumulation of PFL transcripts and an increased production of formate and acetyl-CoA. The decrease in PFL transcripts at five hours post-anaerobiosis could possibly be explained by the increase in formate accumulation acting as a negative feedback to PFL gene transcription. PFL proteins present up to that point may still be active. This hypothetical model displays the functional compensation of fermentation pathways in *C*. *reinhardtii* similarly shown in other studies [29,34].

While it is clear that *HCP4* silencing affects the transcript levels of central fermentation pathways, as well as metabolite production in *C*. *reinhardtii*, further work is needed to fully characterize HCP4’s role in anaerobic metabolism. Transcript abundance changes drastically upon silencing of *HCP4*, but previous studies have shown that PFL and HYD are regulated not only at the transcriptional level, but are also activated post-transcriptionally [29,34]. Further investigation of protein levels in wild-type and *ami-hcp4* would further guide understanding of the plasticity of these fermentation pathways.

Unfortunately, H_2_ gas evolution was not measured in this study due to technical difficulties. Thus the results presented here provide an incomplete picture of metabolic flux during anoxia. Quantifying H_2_ production in *C*. *reinhardtii* in *ami-hcp4* and wild-type would further support or reject the hypothesis that HCP4 competes with HYD for electrons from ferredoxin, as well as the extent to which silencing *HCP4* has in increasing the viability of H_2_ production via *C*. *reinhardtii* at a commercial level. In vitro experiments with isolated ferredoxin and HCP4 could be used examine their potential interactions, such as seen in Wolfe *et al*. 2002 [17]. Following these experiments, a more global examination of metabolites (including quantification of H_2_ production using mass-spec and total starch breakdown) would generate the data needed to pinpoint HCP4’s position in *C*. *reinhardtii* fermentation, as well as the potential HCP4 mutants have for increasing the production of biofuels. It is also imperative to address potential functional redundancy between HCP4 and the other three HCPs encoded by the *C*. *reinhardtii* genome, which all share extensive sequence similarity (S1 Fig).

The drastic downregulation of *PDC* was an unexpected and interesting finding. Although PDC and hydrogenase seem to have similar expression patterns (being upregulated during oxidative stress and anaerobiosis) their direct link is unclear. These results strongly suggest HCP4 somehow regulates the expression of the PDC. PDC expression was shown in other organisms to be dependent on intracellular pH. In this experiment cultures were heavily buffered at pH 7.5. It would be interesting to see whether intracellular or extracellular pH changes occur upon silencing of HCP4 in a less heavily buffered media.

While *HCP4* is most highly upregulated during anaerobiosis, it is also up-regulated during sulfur deprivation-induced anaerobiosis, which creates much more H_2_ gas than darkness-induced anaerobiosis [35]. If the hypothesis that *HCP4* oxidizes reduced ferredoxin is supported, then the increase in H_2_ output could be drastically increased in these cultures. Therefore investigation of *ami-hcp4* mutants must be examined in these conditions in order to determine *HCP4*’s potential impact on the economic feasibility of H_2_ production in *C*. *reinhardtii*.

## Conclusions

This study demonstrates *C*. *reinhardtii* fermentative metabolism is not only phylogenetically diverse and functionally plastic, but that reverse genetic techniques can be applied to increase biofuel production. For example, down-regulating *HCP4* resulted in a significant increase in ethanol production. The increased ethanol, and potentially increased H_2_ production rates, in this mutant may have sizeable benefits to mass production of biofuels in *C*. *reinhardtii*. Its potential role in nitrogen metabolism may also be an important avenue of future investigation.

## Supporting Information

S1 Fig CLUSTAL Omega (1.2.1) multiple sequence alignment of HCPs. (DOCX)

S1 Table Oligonucleotides for creating insert encoding amiRNA targeting *HCP4*. (DOCX)

S2 Table Oligonucleotide primers used for qRT-PCR. (DOCX)

S3 Table Sequences of HCPs used for alignment shown in S1 Fig. (DOCX)

## Abbreviations

PFL: pyruvate formate lyase
PDC: pyruvate decarboxylase
PFR: pyruvate ferredoxin oxidoreductase
LDH: lactate dehydrogenase
HYD: hydrogenase
ADH: alcohol dehydrogenase
PAT1/PAT2: phosphoacetyl transferase
ACK1/ACK2: acetate kinase
NR: nitrate reductase
NiR: nitrite reductase
GS/GOGAT: glutamine sythetase/glutamate synthase cycle
GOGAT: glutamine oxoglutarate amidotransferase
TAP: Tris acetate phosphate media
amiRNA: artificial microRNA
ami-hcp4: transgenic *C*. *reinhardtii* containing amiRNA targeting HCP4
HCP: hybrid cluster protein
HCP4: hybrid cluster protein 4
CAIP: calf intestinal alkaline phosphatase
