# Supplementary material for: The Involvement of hybrid cluster protein 4, HCP4, in Anaerobic Metabolism in Chlamydomonas reinhardtii
Source: PLoS One. 2016 Mar 1;11(3):e0149816. doi: 10.1371/journal.pone.0149816 (PMC4773151; doi:10.1371/journal.pone.0149816)
Supplement: S1 Fig — (DOCX) [file pone.0149816.s001.docx]

**Supporting Figure 1.** CLUSTAL O (1.2.1) multiple sequence alignment of HCPs.

EcHCP ------------------------------------------------------------ 0

CrHCP1 -------MLRAPVMSSAASRKAAVPAVAARAGCRRVGAMRVFAFQKST--G-SVHDK--N 48

CrHCP4 -------MLRAPVMSSAASRKVAAPAVAARAGCRRVGVMRVFAFQKAASCD-NLHDK--N 50

CrHCP2 MLSRCLGMAGTTLGGSLASGAQSAVSGMFRASGRRATSLQVLAWQLPNLFAGDQQARNAA 60

CrHCP3 MLARCMSMASTTVRGHLVRGSSGSLSAS---VARGTGAVRVMAWEW-NPFAGDQQARD-A 55

EcHCP ------------------MFCVQCEQTIRTPAGNGCSYAQGMCGKTAETSDLQDLLIAAL 42

CrHCP1 ALHERIQKSKELLDAESAMMCYQCEQTK---SGTGCT-DIGVCGKTPEVSALQDLLIYSV 104

CrHCP4 ALHERIQKSKELLDAESAMMCYQCEQTK---SGTGCT-DIGVCGKTPEVSALQDLLIYSV 106

CrHCP2 SIKAKMAEANKA-LESDKMLCYQCEQTK---SGTGCT-EIGVCGKTPEVAGLQDLLVYSV 115

CrHCP3 SIKAKMAEANKA-LEHDKMLCYQCEQTK---SGTGCT-EIGVCGKTPEVAGLQDLLVYSV 110

*:* ***** :*.**: *:**** *.: *****: ::

EcHCP QGLSAWAVKARE--YGIINHDVDSFAPRAFFSTLTNVNFDSPRIVGYAREAIALREALKA 100

CrHCP1 KGLGSLAHVARTSPAKIEDAAVNTFINGAIFSTLTNVNFADDRFLEFVTDCRKLHAQLAA 164

CrHCP4 KGLGSLAHVARTSPAKIEDAAVNTFINGAIFSTLTNVNFADDRFLEFVTDCRKLHAQLAA 166

CrHCP2 KGLASLAHIARNSPAKIEDPAVNTFINGAIFSTLTNVNFADDRFLEFVSEARAHHARLSA 175

CrHCP3 KGLASLAHIARNSPAKIEDPAVNTFINGAIFSTLTNVNFADDRFLEFVSEARAHHARLSA 170

:**.: * ** * : *::* *:********* . *:: :. :. : * *

EcHCP QCLAVD----ANARVDN--------PMA---DLQLVSDDLGELQRQAAEFTPNKDKAAIG 145

CrHCP1 KMAAAGVAVPAAETAHQPWFGSMPHPLAWNSDTHVALAGVGDMLEVASKTGVKERQHVLG 224

CrHCP4 KMAAAGVAVPAAETAHQPWFGSMPHPLAWNSDTHVALAGVGDMLEVASKTGVKERQHVLG 226

CrHCP2 KMAAAGVQVPASATEQQVWFGSMPHPLLWNSQA-AALGGVGDMLEVAAKTGIAERQKVLG 234

CrHCP3 KMAAAGVQVPASATEQQVWFGSMPHPLLWNSQA-AALGGVGDMLEVAAKTGIAERQRVLG 229

: *. * .: *: : . :*:: . *:: : : .:*

EcHCP ENILGLRLLCLYGLKGAAAYMEHAHVLGQYDNDIYAQYHKIMAWLGTW-PADMNALLECS 204

CrHCP1 ETLAGLQELLMYGLKGLCAYAHHAEALGHTDPAVYADVQAYLHFLCSPAAADVGQVLDAC 284

CrHCP4 ETLAGLQELLMYGLKGLCAYAHHAEALGHTDPAVYADVQAYLHFLCSPAAADVGQVLDAC 286

CrHCP2 ETLAGLQELLVYGLKGVCAYAHHAEALGFTDPTVYAEIQGALHFLNTPGAKDVGQVLDAC 294

CrHCP3 ETLAGLQELLVYGLKGVCAYAHHAEALGFTDPTVYAEIQGALHFLNTPGAKDVGQVLDAC 289

*.: **: * :***** .** .**..** * :**: : : :* : *: :*:..

EcHCP MEIGQMNFKVMSILDAGETGKYGHPTPTQVNVKATAGKCILISGHDLKDLYNLLEQTEGT 264

CrHCP1 FRAGATNFRVMEMLSNAHTDTFGHPVPTPVTLNPVPGKAILVTGHDMHDLHMLLEQTAGK 344

CrHCP4 FRAGATNFRVMEMLSNAHTDTFGHPVPTPVTLNPVPGKAILVTGHDMHDLHMLLEQTAGK 346

CrHCP2 FKCGATNFKVMEMLSNAHTDTFGHPVPTPVTLNPVPGKAILVTGHDMHDLHMLLEQTAGK 354

CrHCP3 FKCGATNFKVMEMLSNAHTDTFGHPVPTPVTLNPVPGKAILVTGHDMHDLHMLLEQTAGK 349

:. * **:**.:*. ..* .:***.** *.:: . **.**::***::**: ***** *.

EcHCP GVNVYTHGEMLPAHGYPELRKFKHLVGNYGSGWQNQQVEFARFPGPIVMTSNCIIDPTVG 324

CrHCP1 GINVYTHGEMLPAHGYPGLKKYPHLVGHFGGAWYRQKIDFAAFPGAVAVTTNCVLDPL-T 403

CrHCP4 GINVYTHGEMLPAHGYPGLKKYPHLVGHFGGAWYRQKIDFAAFPGAVAVTTNCVLDPL-T 405

CrHCP2 GINVYTHGEMLPAHGYPGLKKYPHLVGHFGGAWYRQKIDFAEFPGAVAVTTNCVLDPL-Q 413

CrHCP3 GINVYTHGEMLPAHGYPGLKKYPHLVGHFGGAWYRQKIDFAEFPGAVAVTTNCVLDPL-Q 408

*:*************** *:*: ****.:*..* .*:::** *** :.:*:**::**

EcHCP AYDDRIWTRSIVGWPGVRHLD-----GEDFSAVIAQAQQMAGFPYSEI-----PHLITVG 374

CrHCP1 AYKDNIFTINETGLSGVPHIRPDANGHKDFTPIINRAMQLPGFTAESVAKMEKKRDVTVG 463

CrHCP4 AYKDNIFTINETGLSGVPHIRPDATGHKDFTPIINRALQLPGFTPESVAKMEKKRDVTVG 465

CrHCP2 VYKQNIFTINETGLSGVPHIRPDANGHKDFTPIINRALQLPGFTPELIEKRPKKKDVTVG 473

CrHCP3 VYKQNIFTINETGLSGVPHIRPDANGHKDFTPIINRALQLPGFTPELIEKRPKKKDVTVG 468

.*.:.*:* . .* ** *: :**: :* :* *: ** . : : :***

EcHCP FGRQTLLGAADTLIDLVSREKLRHIFLLGGCDGARGERHYFTDFATSVPDDCLILTLACG 434

CrHCP1 FGHKSVLSVAPQVIQAIQEKRLEHIFLVGGCDGSEPQRKYYSKLYQFMPTNTMVLTLGCG 523

CrHCP4 FGHKSVLSVAPQVIQAIQEKRLEHIFLVGGCDGSEPQRKYYSKLYQFMPTNTMVLTLGCG 525

CrHCP2 FGHKAVLSVAPQVIQAIQEKRLEHIFLVGGCDGSEPQRKYYSKLYQYMPTNTMVLTLGCG 533

CrHCP3 FGHKAVLSVAPQVIQAIQEKRLEHIFLVGGCDGSEPQRKYYSKLYQYMPTNTMVLTLGCG 528

**::::*..* :*: :..::*.****:*****:. :*:*::.: :* : ::***.**

EcHCP KYRFNKLEFGDIEG--LPRLVDAGQCNDAYSAIILAVTLAEKLGCGVNDLPLSLVLSWFE 492

CrHCP1 KFRIFDQDFGTLPGTDLPRLLDMGQCNDAYSALVVATELAKVFKTDVNSLPLSLDLSWFE 583

CrHCP4 KFRIFDQDFGTLPGTDLPRLLDMGQCNDAYSALVVATELAKVFKTDVNSLPLSLDLSWFE 585

CrHCP2 KFRIFDQDFGTLPGTDLPRLLDMGQCNDSYSALVVATELAKVFKTDVNSLPLSLDLSWFE 593

CrHCP3 KFRIFDQDFGTLPGTDLPRLLDMGQCNDSYSALVVATELAKVFKTDVNSLPLSLDLSWFE 588

*:*: . :** : * ****:* *****:***:::*. **: : **.***** *****

EcHCP QKAIVILLTLLSLGVKNIVTGPTAPGFLTPDLLAVLNEKFGLRSITTVE--EDMKQLLSA 550

CrHCP1 QKAVAVLLTLLHLGVRNIRLGPRLPAFLTPEAVGVLVDRFNLIPANVADPGADMKMMMEC 643

CrHCP4 QKAVAVLLTLLHLGVRNIRLGPRLPAFLTPEAVGVLVDRFGLIPANVADPAADMQMMMEC 645

CrHCP2 QKAVAVLLTLLHLGVRNIRLGPRLPAFLTPEAVGVLVDRFNLIPANVADPGADMKMMMKN 653

CrHCP3 QKAVAVLLTLLHLGVRNIRLGPRLPAFLTPEAVGVLVEKFNLIPANVADPGADMKMMMKN 648

***:.:***** ***:** ** *.****: :.** ::* * ...: **: ::.

EcHCP - 550

CrHCP1 K 644

CrHCP4 K 646

CrHCP2 K 654

CrHCP3 K 649
