# Supplementary material for: The Involvement of hybrid cluster protein 4, HCP4, in Anaerobic Metabolism in Chlamydomonas reinhardtii
Source: PLoS One. 2016 Mar 1;11(3):e0149816. doi: 10.1371/journal.pone.0149816 (PMC4773151; doi:10.1371/journal.pone.0149816)
Supplement: S1 Table — (DOCX) [file pone.0149816.s002.docx]

**Supporting Table 1.** Oligonucleotides for creating insert encoding amiRNA targeting *HCP4*.

Forward 5’CTAGTGAGAGTAGTACGTTTAAAATATCTCGCTGATCGGCACCATGGGGGTGGTGGTGATCAGCGCTATATTATAAACGTACTACTCTCG-3’

Reverse

5’CTAGCGAGAGTAGTACGTTTATAATATAGCGCTGATCACCACCACCCCCATGGTGCCGATCAGCGAGATATTTTAAACGTACTACTCTCA -3’
