# Supplementary material for: The Involvement of hybrid cluster protein 4, HCP4, in Anaerobic Metabolism in Chlamydomonas reinhardtii
Source: PLoS One. 2016 Mar 1;11(3):e0149816. doi: 10.1371/journal.pone.0149816 (PMC4773151; doi:10.1371/journal.pone.0149816)
Supplement: S2 Table — (DOCX) [file pone.0149816.s003.docx]

| **Supporting Table 2.** Oligonucleotide primers used for qRT-PCR | | | |
| --- | --- | --- | --- |
| Name | Protein ID | Sequence | Tm |
| Rack1 | 105734 | Forward 5'-CTTCTCGCCCATGACCAC-3' | 55.9°C |
|  |  | Reverse 5'-CCCACCAGGTTGTTCTTCAG-3' | 55.8°C |
| HYD1 | 183963 | Forward 5'-GTCTATTCGCGGCAGCTC-3' | 56.1°C |
|  |  | Reverse 5'-TGCTGGACATGACTCAAAGG-3' | 55°C |
| PFR1 | 122198 | Forward 5'-GTCCGACGTGTCCTTCATCT-3' | 56.6°C |
|  |  | Reverse 5'-ACGGACATGACGTTGTTGAA-3' | 54.7°C |
| PFL1 | 146801 | Forward 5'-ATGTACGCGAACACCATGAA-3' | 54.6°C |
|  |  | Reverse 5'-GTCACCTGGGCGTACTTGAT-3' | 54.7°C |
| PDC1 | 127786 | Forward 5'-TACTCCACTGCCGGCTACTC-3' | 58.6°C |
|  |  | Reverse 5'-AGAGCCATGCGCTTGTAGAT-3' | 56.6°C |
| HCP4 | 148255 | Forward 5'-CCATGATGTGCTACCAGTGC-3' | 56.2°C |
|  |  | Reverse 5'-CCATGATGTGCTACCAGTGC-3' | 54.1°C |
