# Supplementary material for: The Involvement of hybrid cluster protein 4, HCP4, in Anaerobic Metabolism in Chlamydomonas reinhardtii
Source: PLoS One. 2016 Mar 1;11(3):e0149816. doi: 10.1371/journal.pone.0149816 (PMC4773151; doi:10.1371/journal.pone.0149816)
Supplement: S3 Table — (DOCX) [file pone.0149816.s004.docx]

**Supporting Table 3.** Sequences of HCPs used for alignment shown in Supporting Figure 1.

>CrHCP1 (XP_001694571)

MLRAPVMSSAASRKAAVPAVAARAGCRRVGAMRVFAFQKSTGSVHDKNALHERIQKSKELLDAESAMMCYQCEQTKSGTGCTDIGVCGKTPEVSALQDLLIYSVKGLGSLAHVARTSPAKIEDAAVNTFINGAIFSTLTNVNFADDRFLEFVTDCRKLHAQLAAKMAAAGVAVPAAETAHQPWFGSMPHPLAWNSDTHVALAGVGDMLEVASKTGVKERQHVLGETLAGLQELLMYGLKGLCAYAHHAEALGHTDPAVYADVQAYLHFLCSPAAADVGQVLDACFRAGATNFRVMEMLSNAHTDTFGHPVPTPVTLNPVPGKAILVTGHDMHDLHMLLEQTAGKGINVYTHGEMLPAHGYPGLKKYPHLVGHFGGAWYRQKIDFAAFPGAVAVTTNCVLDPLTAYKDNIFTINETGLSGVPHIRPDANGHKDFTPIINRAMQLPGFTAESVAKMEKKRDVTVGFGHKSVLSVAPQVIQAIQEKRLEHIFLVGGCDGSEPQRKYYSKLYQFMPTNTMVLTLGCGKFRIFDQDFGTLPGTDLPRLLDMGQCNDAYSALVVATELAKVFKTDVNSLPLSLDLSWFEQKAVAVLLTLLHLGVRNIRLGPRLPAFLTPEAVGVLVDRFNLIPANVADPGADMKMMMECK

>CrHCP2 (XP_001694756)

MLSRCLGMAGTTLGGSLASGAQSAVSGMFRASGRRATSLQVLAWQLPNLFAGDQQARNAASIKAKMAEANKALESDKMLCYQCEQTKSGTGCTEIGVCGKTPEVAGLQDLLVYSVKGLASLAHIARNSPAKIEDPAVNTFINGAIFSTLTNVNFADDRFLEFVSEARAHHARLSAKMAAAGVQVPASATEQQVWFGSMPHPLLWNSQAAALGGVGDMLEVAAKTGIAERQKVLGETLAGLQELLVYGLKGVCAYAHHAEALGFTDPTVYAEIQGALHFLNTPGAKDVGQVLDACFKCGATNFKVMEMLSNAHTDTFGHPVPTPVTLNPVPGKAILVTGHDMHDLHMLLEQTAGKGINVYTHGEMLPAHGYPGLKKYPHLVGHFGGAWYRQKIDFAEFPGAVAVTTNCVLDPLQVYKQNIFTINETGLSGVPHIRPDANGHKDFTPIINRALQLPGFTPELIEKRPKKKDVTVGFGHKAVLSVAPQVIQAIQEKRLEHIFLVGGCDGSEPQRKYYSKLYQYMPTNTMVLTLGCGKFRIFDQDFGTLPGTDLPRLLDMGQCNDSYSALVVATELAKVFKTDVNSLPLSLDLSWFEQKAVAVLLTLLHLGVRNIRLGPRLPAFLTPEAVGVLVDRFNLIPANVADPGADMKMMMKNK

>CrHCP3 (XP_001694671)

MLARCMSMASTTVRGHLVRGSSGSLSASVARGTGAVRVMAWEWNPFAGDQQARDASIKAKMAEANKALEHDKMLCYQCEQTKSGTGCTEIGVCGKTPEVAGLQDLLVYSVKGLASLAHIARNSPAKIEDPAVNTFINGAIFSTLTNVNFADDRFLEFVSEARAHHARLSAKMAAAGVQVPASATEQQVWFGSMPHPLLWNSQAAALGGVGDMLEVAAKTGIAERQRVLGETLAGLQELLVYGLKGVCAYAHHAEALGFTDPTVYAEIQGALHFLNTPGAKDVGQVLDACFKCGATNFKVMEMLSNAHTDTFGHPVPTPVTLNPVPGKAILVTGHDMHDLHMLLEQTAGKGINVYTHGEMLPAHGYPGLKKYPHLVGHFGGAWYRQKIDFAEFPGAVAVTTNCVLDPLQVYKQNIFTINETGLSGVPHIRPDANGHKDFTPIINRALQLPGFTPELIEKRPKKKDVTVGFGHKAVLSVAPQVIQAIQEKRLEHIFLVGGCDGSEPQRKYYSKLYQYMPTNTMVLTLGCGKFRIFDQDFGTLPGTDLPRLLDMGQCNDSYSALVVATELAKVFKTDVNSLPLSLDLSWFEQKAVAVLLTLLHLGVRNIRLGPRLPAFLTPEAVGVLVEKFNLIPANVADPGADMKMMMKNK

>CrHCP4 (XP_001694454)

MLRAPVMSSAASRKVAAPAVAARAGCRRVGVMRVFAFQKAASCDNLHDKNALHERIQKSKELLDAESAMMCYQCEQTKSGTGCTDIGVCGKTPEVSALQDLLIYSVKGLGSLAHVARTSPAKIEDAAVNTFINGAIFSTLTNVNFADDRFLEFVTDCRKLHAQLAAKMAAAGVAVPAAETAHQPWFGSMPHPLAWNSDTHVALAGVGDMLEVASKTGVKERQHVLGETLAGLQELLMYGLKGLCAYAHHAEALGHTDPAVYADVQAYLHFLCSPAAADVGQVLDACFRAGATNFRVMEMLSNAHTDTFGHPVPTPVTLNPVPGKAILVTGHDMHDLHMLLEQTAGKGINVYTHGEMLPAHGYPGLKKYPHLVGHFGGAWYRQKIDFAAFPGAVAVTTNCVLDPLTAYKDNIFTINETGLSGVPHIRPDATGHKDFTPIINRALQLPGFTPESVAKMEKKRDVTVGFGHKSVLSVAPQVIQAIQEKRLEHIFLVGGCDGSEPQRKYYSKLYQFMPTNTMVLTLGCGKFRIFDQDFGTLPGTDLPRLLDMGQCNDAYSALVVATELAKVFKTDVNSLPLSLDLSWFEQKAVAVLLTLLHLGVRNIRLGPRLPAFLTPEAVGVLVDRFGLIPANVADPAADMQMMMECK

>EcHCP (EGT67064)

MFCVQCEQTIRTPAGNGCSYAQGMCGKTAETSDLQDLLIAALQGLSAWAVKAREYGIINHDVDSFAPRAFFSTLTNVNFDSPRIVGYAREAIALREALKAQCLAVDANARVDNPMADLQLVSDDLGELQRQAAEFTPNKDKAAIGENILGLRLLCLYGLKGAAAYMEHAHVLGQYDNDIYAQYHKIMAWLGTWPADMNALLECSMEIGQMNFKVMSILDAGETGKYGHPTPTQVNVKATAGKCILISGHDLKDLYNLLEQTEGTGVNVYTHGEMLPAHGYPELRKFKHLVGNYGSGWQNQQVEFARFPGPIVMTSNCIIDPTVGAYDDRIWTRSIVGWPGVRHLDGEDFSAVIAQAQQMAGFPYSEIPHLITVGFGRQTLLGAADTLIDLVSREKLRHIFLLGGCDGARGERHYFTDFATSVPDDCLILTLACGKYRFNKLEFGDIEGLPRLVDAGQCNDAYSAIILAVTLAEKLGCGVNDLPLSLVLSWFEQKAIVILLTLLSLGVKNIVTGPTAPGFLTPDLLAVLNEKFGLRSITTVEEDMKQLLSA
